# Reduction of Orbital Inflammation following Decompression for Thyroid-Related Orbitopathy

**DOI:** 10.1155/2013/794984

**Published:** 2013-06-18

**Authors:** Sang-Rog Oh, Jonathan D. Tung, Ayelet Priel, Leah Levi, David B. Granet, Bobby S. Korn, Don O. Kikkawa

**Affiliations:** ^1^Division of Oculofacial Plastic and Reconstructive Surgery, UCSD Department of Ophthalmology, CA, USA; ^2^Thyroid Eye Center, UCSD Department of Ophthalmology, La Jolla, CA, USA; ^3^Division of Oculofacial Plastic and Reconstructive Surgery, Department of Ophthalmology, The Permanente Medical Group, Sacramento, CA, USA; ^4^UCSD Department of Ophthalmology, La Jolla, CA, USA; ^5^UCSD Department of Neurosciences, La Jolla, CA, USA; ^6^Division of Pediatric Ophthalmology, UCSD Department of Ophthalmology, CA, USA

## Abstract

*Purpose*. Thyroid-related orbitopathy (TRO) is associated with inflammation, expansion of orbital fat, enlargement of extraocular muscles, and optic neuropathy (ON). We examined the effects of orbital decompression on the inflammatory and congestive signs of TRO in patients who underwent emergent orbital decompression. *Methods*. This retrospective, consecutive study included patients with ON from TRO who underwent orbital decompression. Pre- and postoperative orbital inflammatory signs in the operated and nonoperated, contralateral eyes were graded with the 10-item clinical activity score (CAS). *Results*. Thirty-one orbits were included. Postoperatively, 22 patients and 29 orbits had resolution of ON while the remaining 2 patients had improvement in visual acuity. Mean preoperative CAS was 9.5 ± 0.4. At 12 months, postoperative CAS was 2.1 ± 0.6 (*P* < 0.01) in the operated eye and 3.2 ± 0.5 (*P* < 0.05) in the nonoperated, contralateral eye. *Conclusion*. In our series, 94% of orbits had resolution of ON. There was also a statistically significant postoperative reduction in the CAS in both the operated and nonoperated, contralateral eyes. This phenomenon may be due to lowered venous congestion, decreased intraorbital pressure, and diminution in inflammatory factors.

## 1. Introduction

Thyroid-related orbitopathy (TRO) affects approximately fifty percent of patients with thyroid dysfunction [1]. Current understanding of the pathophysiology of TRO suggests that dysregulation of orbital fibroblasts leads to the cellular changes that are responsible for enlargement of extraocular muscles and expansion of orbital fat [2]. However, the exact pathophysiology of TRO has not been clearly elucidated. The active phase of the disease, which usually lasts from six to twelve months, is characterized by discomfort, eyelid retraction, proptosis, eyelid and conjunctival edema, exposure keratopathy, restrictive strabismus, and, rarely, optic neuropathy (ON) [3].

Humoral and cell-mediated immune processes are likely involved and result from cross-reactivity against shared antigens in both thyroid and orbital tissues, causing immune-mediated inflammation [4–6]. Based on this understanding, immunomodulatory therapy aimed at reducing the active inflammatory phase of the disease has been advocated. Corticosteroids [7] and external beam orbital radiation [8] (XRT) are commonly used agents for TRO, with newer modalities, such as rituximab (RTX), being investigated [9, 10]. Presently, there is inadequate evidence to ascertain whether medical treatment reliably shortens the active phase of TRO, and there is no agreement on the standard therapeutic protocol for the treatment of TRO [11, 12].

For symptomatic patients without sight-threatening disease, orbital decompression is indicated after the active inflammatory phase has passed, and the patient's disease has stabilized [13]. In three to five percent of cases, compressive ON and vision-threatening corneal exposure can be seen, requiring prompt treatment [14, 15]. It is thought that optic nerve compression at the orbital apex from enlarged extraocular muscles, increased inflammation, and orbital fat hypertrophy are responsible for ON [16]. Some have suggested that ON persisting after medical and surgical therapies may be due to optic nerve ischemia or optic neuritis [17, 18]. We previously described ON after botulinum toxin administration for restrictive myopathy which was reversed after corticosteroid therapy followed by prompt orbital decompression [19]. Although corticosteroids, XRT, and orbital decompression have all been shown to be beneficial, there is no universal agreement in the sequence or efficacy of treatment for ON from TRO [20].

Numerous studies have examined the visual outcome after orbital decompression in patients with ON [21–34]. Several studies have noted CAS and inflammatory indices following surgical and medical treatment for ON, but they were not the studied in depth [35, 36]. We present a series of patients who underwent expeditious orbital decompression for ON. The goal of this study was to compare the effects of surgery on not only visual recovery, but also disease activity status.

## 2. Materials and Methods

We retrospectively reviewed the medical records of all patients who underwent orbital decompression surgery for ON from TRO, between 2000 and 2010, at the University of California, San Diego, Shiley Eye Center. The UCSD Human Research Institutional Review Board approval was obtained. We analyzed these patients' records and extracted information regarding demographic characteristics, thyroid status, and previous medical treatments, as well as relevant ophthalmic findings including pre- and postoperative diplopia and the type of orbital decompression performed. Exclusion criteria included previous eyelid or orbital surgery, any history of previous unrelated optic nerve disease, and decreased visual acuity from other causes. 

### 2.1. Optic Neuropathy

ON was diagnosed on evidence of decreased visual acuity along with color vision abnormality on Ishihara (Ishihara Medical Supplies) or Hardy-Rand-Rittler (Richmond Products, Albuquerque, NM, USA) color plates, a visual field defect not related to other ophthalmic diseases, or a relative afferent pupillary defect in unilateral or asymmetric cases. All cases had orbital apex crowding on imaging studies, confirming that the optic neuropathy was related to their TRO. 

### 2.2. Orbital Inflammation

Preoperatively, patients were examined for proptosis, lid retraction, restrictive strabismus, conjunctival injection, chemosis, periocular erythema, pain, and edema. They were then graded with the 10-item CAS. At three, six, and twelve months of followup, postoperative CAS in both the operated and nonoperated contralateral eyes was again assessed.

### 2.3. Orbital Decompression

All patients underwent maximal decompression ± removal of the bony lateral rim as previously described (DOK and BSK) [37–39]. Orbital fat decompression was performed as well. Two milliliters of peribulbar steroid (1 : 1 mixture of triamcinolone acetonide 40 mg/mL and methylprednisolone sodium succinate 125 mg/mL) was administered at the time of surgery. 

### 2.4. Outcome Measures

Improvement of optic neuropathy was determined by improvement in visual acuity by 2 or more lines, resolution of color vision abnormality, resolution of visual field defects, and resolution of relative afferent pupillary defect. Pre- and postoperative CAS were compared. 

Statistical analysis was carried out using SPSS 14.0 (SPSS, Inc., Chicago, IL, USA). Statistical significance was set at *P* < 0.05.

## 3. Results

Twenty-four patients underwent orbital decompression for ON. Seven patients had bilateral ON. Average age was 55.1 ± 14.3 years. The cohort included 7 men and 17 women. All 24 patients had decreased visual acuity compared to their previous baseline examination, 15 patients had a relative afferent pupillary defect, and 13 patients had color deficiency. All 24 patients had worsening visual field deficits, when compared to baseline testing, and optic nerve edema on dilated funduscopic examination. 

All 24 patients underwent orbital decompression within 7 days of presentation. Each orbit in the bilateral cases was considered separately. In all, 31 orbits were included in the study. Ten patients were treated elsewhere with either intravenous or oral corticosteroids and were taking corticosteroids when they presented to the Thyroid Eye Center. They were treated with corticosteroids for an average of 8.4 weeks prior to orbital decompression. The other 14 patients began treatment with oral corticosteroids after their initial visit at the Thyroid Eye Center for ON. These patients were treated for an average of 4.6 days prior to orbital decompression. Following orbital decompression, oral corticosteroids were tapered within 7 days in all 24 patients. None of the patients had reactivation of their TRO after surgery. 23 patients had pre-operative diplopia. Two patients had resolution of their diplopia after decompression. One patient had no diplopia before and after surgery. 

**Figure 1:**
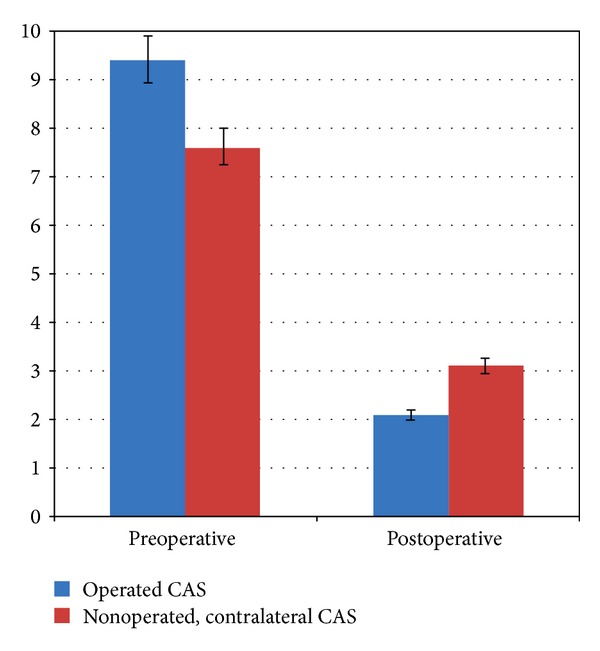
Preoperative and postoperative clinical activity scores (CAS) on the operated eye and nonoperated, contralateral eye at 12 months following orbital decompression.

At 12 months, 29 orbits (94%) in 22 patients (92%) had resolution of ON. The remaining 2 patients (8%), who had unilateral ON, had greater than 2 lines of improvement in visual acuity, but had residual color vision and visual field deficits. Average reduction in exophthalmos after surgery was 6.2 ± 1.7 mm. Preoperative CAS was 9.5 ± 0.4. At 6 months, postoperative CAS in the operated eye was 2.9 ± 0.4  (*P* < 0.01), and at 12 months, postoperative CAS in the operated eye was 2.1 ± 0.6  (*P* < 0.01) (Figure 1). In the nonoperated, contralateral eye, preoperative CAS was 7.4 ± 0.3. At 6 months, postoperative CAS in the non-operated, contralateral eye was 3.5 ± 0.1  (*P* < 0.05), and at 12 months, postoperative CAS in the non-operated, contralateral eye was 3.2 ± 0.5  (*P* < 0.05) (Figure 1).

Ten patients had been treated with corticosteroids for an average of 8.4 weeks when they presented to the Thyroid Eye Center. Their preoperative CAS of 8.3 ± 0.6 and their post-operative CAS was 2.0 ± 0.3  (*P* < 0.01). This was not statistically different from the other 14 patients, who were treated with corticosteroids for 4.6 days prior to orbital decompression. 

### 3.1. Selected Cases

**Case 1**

**Figure 2:**
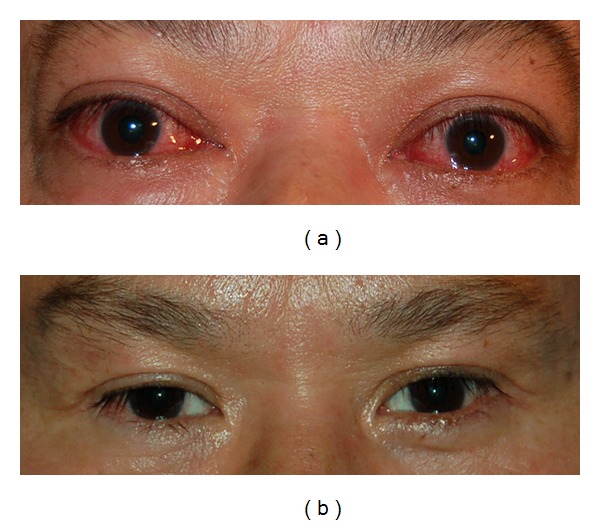
(a) A 50-year-old male with bilateral optic neuropathy with CAS 9 in both eyes. (b) Six months after bilateral orbital decompression with resolution of optic neuropathy and CAS 2 in both eyes.

A 50-year-old male presented with bilateral optic neuropathy. His visual acuity was 20/60 in both eyes with impaired color vision (Figure 2(a)). His pre-operative CAS was 9 in both eyes. Six months after bilateral orbital decompression, his visual acuity was 20/20 in both eyes with full color vision (Figure 2(b)).  His post-operative CAS was 2 in both eyes at 6 months of followup.

**Case 2**

**Figure 3:**
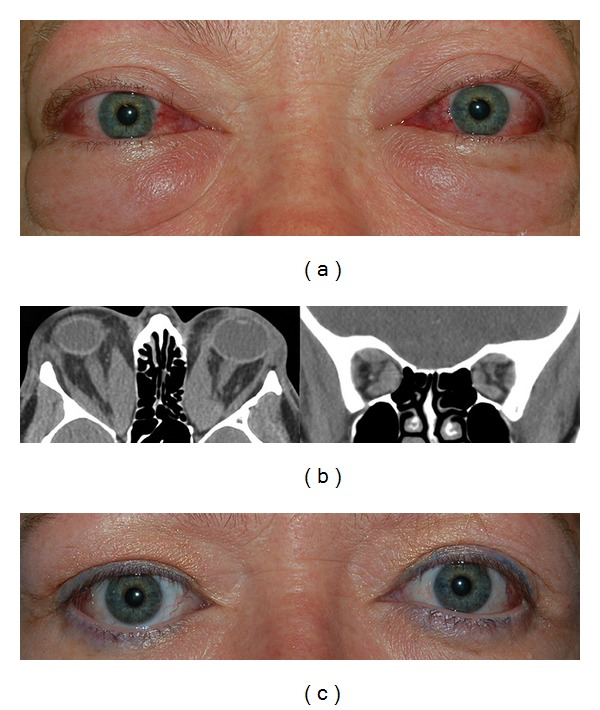
(a) A 49-year-old female who failed intravenous and oral corticosteroids with bilateral optic neuropathy and CAS 9 in the right eye and 10 in the left eye. (b) Computed tomography of her orbits showed enlarged extraocular muscles and orbital apex crowding. (c) 12 weeks after bilateral orbital decompression with resolution of optic neuropathy and CAS 2 in both eyes.

A 49-year-old female had been previously treated elsewhere with intravenous pulsed corticosteroids six months prior to presentation and was taking oral corticosteroids when she was first examined at the Thyroid Eye Center. Her visual acuity was 20/80 in the right eye and 20/70 in the left eye with impaired color vision in both eyes (Figure 3(a)). Computed tomography of her orbits showed enlarged extraocular muscles and orbital apex crowding (Figure 3(b)). Her pre-operative CAS was 9 in the right eye and 10 in the left eye. After bilateral orbital decompression, her vision was 20/20 in both eyes with resolution of color vision defects (Figure 3(c)). Postoperative CAS was 2 in both eyes at 12 weeks after surgery.

**Case 3**

**Figure 4:**
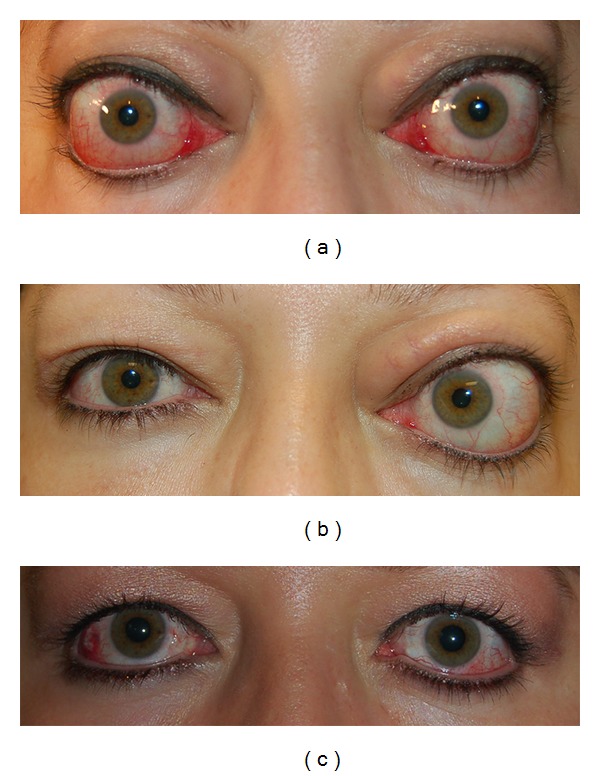
(a) A 47-year-old female with right optic neuropathy. Her preoperative CAS was 10 in the right eye and 9 in the left eye. (b) 8 weeks after unilateral, right orbital decompression, she had resolution of her right optic neuropathy. Her postoperative CAS was 3 in the right eye and 4 in the left eye. (c) 6 weeks after left orbital decompression for disfiguring proptosis, with symmetric exophthalmometry measurements.

A 47-year-old female presented with optic neuropathy in the right eye. Her visual acuity was 20/60 in the right eye and 20/25 in the left eye. She also had impaired color vision in the right eye (Figure 4(a)). Her pre-operative CAS was 10 in the right eye and 9 in the left eye. After right orbital decompression, her visual acuity was 20/25 in the right eye with resolution of optic neuropathy (Figure 4(b)). Her post-operative CAS was 3 in the right eye and 4 in the left eye at 8 weeks of followup. She later had left orbital decompression for disfiguring proptosis, with symmetric exophthalmometry measurements (Figure 4(c)).

## 4. Discussion

Our cohort included 24 patients who had urgent surgical orbital decompression for ON. Ninety-four percent had reversal of ON. Among these patients, the average CAS at presentation was 9.5 ± 0.4. Postoperatively, the average CAS was 2.1 ± 0.6, *P* < 0.01. Patients who had been treated with corticosteroids prior to their presentation to the Thyroid Eye Center for an average of 8.4 weeks prior to their surgical decompression had preoperative CAS of 8.3 ± 0.6. This group also had a statistically significant decrease in CAS after surgery (2.0 ± 0.3). To the authors' best knowledge, this is the first report of a reduction in inflammatory signs and symptoms after urgent surgical decompression for optic neuropathy (keywords: orbital decompression, clinical activity score, inflammation, congestion, Graves' disease, orbitopathy, and optic neuropathy).

After orbital decompression, there was a statistically significant reduction in orbital congestion such as chemosis, pain, and eyelid edema [40]. At 12 months, average CAS decreased from 9.5 to 2.1 (*P* < 0.01) (Figure 1). Ten patients had been treated with chronic corticosteroids prior to their decompression (8.4 weeks). These patients had similar CAS scores pre- and postoperatively, when compared to those who were started on oral corticosteroids for a short period prior to their decompression (4.6 days). We hypothesize that resolution of congestive symptoms brought on by expansion of orbital fat and extraocular muscle hypertrophy may be partially explained by relief of venous stasis. By expanding orbital volume, there is improved venous outflow with subsequent relief of congestion. It may be impossible to distinguish with certainty if the reduction in CAS is from decreased inflammation, reduction in venous congestion or both.

Some reduction in inflammatory signs such as conjunctival injection and erythema of the eyelids, however, cannot be entirely explained by changes in hydrostatic pressure and intraorbital volume. When the CAS was examined in the nonoperated, contralateral eye, we noted a statistically significant reduction from 7.4 to 3.2 (*P* < 0.05) (Figure 1). Orbital fibroblasts and orbital stem cells have been postulated as targets for immunoglobulins in TRO [2, 41]. All patients underwent orbital fat removal during surgery, and perhaps it is this reduction in inciting factors in the operated eye that leads to a decrease in overall inflammatory cascade, with a reduction of inflammation in the contralateral eye. The mechanism may be similar to sympathetic ophthalmia, where there is improvement in the contralateral eye after enucleation of the inciting eye [42]. Another possibility is spontaneous improvement from disease modulation as time progresses (Rundle's curve). Pre- and postoperative inflammatory indices such as erythrocyte sedimentation rate or C-reactive protein were not obtained in our study group. Ultimately, histopathologic studies evaluating the molecular and cellular changes in the orbital contents before and after orbital decompression are necessary to fully elucidate this phenomenon. Lastly, the effect from perioperative oral and intraoperative periorbital steroid effects cannot be ignored.

Surgical decompression of the bony orbit is traditionally advocated for disfiguring proptosis after the inflammatory phase of TRO has stabilized, unless there is optic neuropathy or vision threatening corneal exposure [13, 15]. The deep lateral wall, medial wall, and the orbital floor may all be decompressed, with exquisite attention paid towards maximal apical expansion [19, 43]. Some studies have shown efficacy of performing medial wall decompression for compressive ON [44]. In the current series, all patients underwent maximal orbital decompression of all three walls, as it is our opinion that a maximally decompressed apex gives the best visual outcome and relief of optic nerve crowding, as well as the best chance to reduce orbital congestion. Some have advocated a “balanced orbital decompression” where the medial and lateral walls are removed with retention of the floor to decrease the risk of new onset of diplopia [45]. In our cohort one patient did not have preoperative diplopia and remained free of diplopia postoperatively. Interestingly, two patients had resolution of their diplopia following surgery. The remaining patients had diplopia before and after orbital decompression. 

TRO activity normally follows Rundle's curve where there is an initial deterioration of the symptoms followed by a period of stability and then improvement [46]. From start to finish, this time course is approximately 1 year. Traditionally, definitive intervention is initiated after the disease has stabilized. In this study, we treated ON with maximal decompression prior to disease stabilization and noted stability at one year. The results of our study raise the question whether early intervention for optic neuropathy improves clinical outcome [47].

This study is limited by several factors. First, it is retrospective in nature. A randomized, controlled study comparing medical and surgical therapy for ON in patients with bilateral disease would be ideal, but would be difficult to conduct. Second, histologic examination of orbital tissue before and after orbital decompression may reveal changing inflammatory markers, but may not be feasible as tissue sampling may not be possible. Third, some patients in the cohort were treated with corticosteroids for 8.4 weeks while others were treated for a much shorter duration, prior to orbital decompression. Although they all had similar preoperative CAS and similar statistically significant reduction in CAS after surgery, this represents a confounding variable that cannot be ignored. Fourth, our mean postoperative follow-up period is 12 months. Ideally, a longer follow-up period could be studied to rule out late reactivation of disease. Fifth, although the CAS objectively measures active disease status, it is difficult to differentiate inflammatory from congestive signs. Finally, there were a limited number of patients. A multicenter study with large numbers would be needed to strengthen the findings of the study.

In summary, we describe the resolution of inflammatory and congestive symptoms in patients treated for ON with orbital decompression. Future studies are directed towards longer-term studies both on a prospective basis and aimed towards defining a cellular and molecular basis to explain these findings.

## References

[1] Forbes G, Gorman CA, Brennan MD (1986). Ophthalmopathy of Graves’ disease: computerized volume measurements of the orbital fat and muscle. *American Journal of Neuroradiology*.

[2] Prabhakar BS, Bahn RS, Smith TJ (2003). Current perspective on the pathogenesis of Graves' disease and ophthalmopathy. *Endocrine Reviews*.

[3] Bahn RS (2010). Graves’ ophthalmopathy. *The New England Journal of Medicine*.

[4] Rapoport B, McLachlan SM (2007). The thyrotropin receptor in Graves’ disease. *Thyroid*.

[5] Khoo DH, Eng PH, Ho SC (2000). Graves’ ophthalmopathy in the absence of elevated free thyroxine and triiodothyronine levels: prevalence, natural history, and thyrotropin receptor antibody levels. *Thyroid*.

[6] Davies TF, Teng CS, McLachlan SM, Smith BR, Hall R (1978). Thyrotropin receptors in adipose tissue, retro-orbital tissue and lymphocytes. *Molecular and Cellular Endocrinology*.

[7] Bartalena L, Pinchera A, Marcocci C (2000). Management of graves’ ophthalmopathy: reality and perspectives. *Endocrine Reviews*.

[8] Gorman CA, Garrity JA, Fatourechi V (2001). A prospective, randomized, double-blind, placebo-controlled study of orbital radiotherapy for Graves’ ophthalmopathy. *Ophthalmology*.

[9] Salvi M, Vannucchi G, Campi I (2007). Treatment of graves’ disease and associated opthalmopathy with the anti-CD20 monoclonal antibody rituximab: an open study. *European Journal of Endocrinology*.

[10] El Fassi D, Nielsen CH, Hasselbalch HC, Hegedüs L (2006). The rationale for B lymphocyte depletion in Graves’ disease: monoclonal anti-CD20 antibody therapy as a novel treatment option. *European Journal of Endocrinology*.

[11] Zoumalan CI, Cockerham KP, Turbin RE (2007). Efficacy of corticosteroids and external beam radiation in the management of moderate to severe thyroid eye disease. *Journal of Neuro-Ophthalmology*.

[12] Bartalena L, Baldeschi L, Dickinson AJ (2008). Consensus statement of the European group on Graves’ orbitopathy (EUGOGO) on management of Graves’ orbitopathy. *Thyroid*.

[13] Thaller SR, Kawamoto HK (1990). Surgical correction of exophthalmos secondary to Graves’ disease. *Plastic and Reconstructive Surgery*.

[14] Burch HB, Wartofsky L (1993). Graves’ ophthalmopathy: current concepts regarding pathogenesis and management. *Endocrine Reviews*.

[15] Bahn RS, Bartley GB, Gorman CA (1992). Emergency treatment of Graves’ ophthalmopathy. *Bailliere’s Clinical Endocrinology and Metabolism*.

[16] Neigel JM, Rootman J, Belkin RI (1988). Dysthyroid optic neuropathy. The crowded orbital apex syndrome. *Ophthalmology*.

[17] Hayreh SS, Joos KM, Podhajsky PA, Long CR (1994). Systemic diseases associated with nonarteritic anterior ischemic optic neuropathy. *American Journal of Ophthalmology*.

[18] Kazim M, Goldberg RA, Smith TJ (2002). Insight to the pathogenesis of thyrois associated ophthalmopathy. *Archives of Ophthalmology*.

[19] Korn BS, Seo S-W, Levi L, Granet DB, Kikkawa DO (2007). Optic neuropathy associated with botulinum A toxin in thyroid-related orbitopathy. *Ophthalmic Plastic and Reconstructive Surgery*.

[20] Jacobson DM (2000). Dysthyroid orbitopathy. *Seminars in Neurology*.

[21] Choe CH, Cho RI, Elner VM (2011). Comparison of lateral and medial orbital decompression for the treatment of compressive optic neuropathy in thyroid eye disease. *Ophthalmic Plastic and Reconstructive Surgery*.

[22] Mensah A, Vignal-Clermont C, Chadi M (2009). Dysthyroid optic neuropathy: atypical initial presentation and persistent visual loss. *Orbit*.

[23] Chu EA, Miller NR, Grant MP, Merbs S, Tufano RP, Lane AP (2009). Surgical treatment of dysthyroid orbitopathy. *Otolaryngology*.

[24] Pribitkin EA, McJunkin J, Kung B, Carrasco JR, Bilyk JR, Savino PJ (2009). Technique selection for orbital decompression: combined endoscopic and transconjunctival versus combined endoscopic and transantral approach. *Ear, Nose and Throat Journal*.

[25] Chu EA, Miller NR, Lane AP (2009). Selective endoscopic decompression of the orbital apex for dysthyroid optic neuropathy. *Laryngoscope*.

[26] Dubin MR, Tabaee A, Scruggs JT, Kazim M, Close LG (2008). Image-guided endoscopic orbital decompression for graves’ orbitopathy. *Annals of Otology, Rhinology and Laryngology*.

[27] McKeag D, Lane C, Lazarus JH (2007). Clinical features of dysthyroid optic neuropathy: a European Group on Graves’ Orbitopathy (EUGOGO) survey. *British Journal of Ophthalmology*.

[28] Ben Simon GJ, Syed H, Douglas R, Schwartz R, Goldberg RA, McCann JD (2006). Clinical manifestations and treatment outcome of optic neuropathy in thyroid-related orbitopathy. *Ophthalmic Surgery Lasers and Imaging*.

[29] Liao SL, Chang TC, Lin LL-K (2006). Transcaruncular orbital decompression: an alternate procedure for graves ophthalmopathy with compressive optic neuropathy. *American Journal of Ophthalmology*.

[30] Schaefer SD, Soliemanzadeh P, Rocca DAD (2003). Endoscopic and transconjunctival orbital decompression for thyroid-related orbital apex compression. *Laryngoscope*.

[31] Michel O, Oberlander N, Neugebauer A, Fricke J, Russmann W (2000). Preliminary report: long-term results of transnasal orbital decompression in malignant Graves’ ophthalmopathy. *Strabismus*.

[32] Neugebauer A, Nishino K, Neugebauer P, Konen W, Michel O (1996). Effects of bilateral orbital decompression by an endoscopic endonasal approach in dysthyroid orbitopathy. *British Journal of Ophthalmology*.

[33] Girod DA, Orcutt JC, Cummings CW (1993). Orbital decompression for preservation of vision in Graves’ ophthalmopathy. *Archives of Otolaryngology*.

[34] Hallin ES, Feldon SE, Luttrell J (1988). Graves’ ophthalmopathy: III. Effect of transantral orbital decompression on optic neuropathy. *British Journal of Ophthalmology*.

[35] Wakelkamp IMMJ, Baldeschi L, Saeed P, Mourits MP, Prummel MF, Wiersinga WM (2005). Surgical or medical decompression as a first-line treatment of optic neuropathy in Graves’ ophthalmopathy? A randomized controlled trial. *Clinical Endocrinology*.

[36] Bartelena L, Marcocci C, Bogazzi F (1989). Orbital decompression for severe Graves’ ophthalmopathy. *Journal of Neurosurgical Sciences*.

[37] Garrity JA, Fatourechi V, Bergstralh EJ (1993). Results of transantral orbital decompression in 428 patients with severe Graves’ ophthalmopathy. *American Journal of Ophthalmology*.

[38] Leone CR, Piest KL, Newman RJ (1989). Medial and lateral wall decompression for thyroid ophthalmopathy. *American Journal of Ophthalmology*.

[39] Goldberg RA, Kim AJ, Kerivan KM (1998). The lacrimal keyhole, orbital door jamb, and basin of the inferior orbital fissure: three areas of deep bone in the lateral orbit. *Archives of Ophthalmology*.

[40] Wakelkamp IMMJ, Baldeschi L, Saeed P, Mourits MP, Prummel MF, Wiersinga WM (2005). Surgical or medical decompression as a first-line treatment of optic neuropathy in Graves’ ophthalmopathy? A randomized controlled trial. *Clinical Endocrinology*.

[41] Korn BS, Kikkawa DO, Hicok KC (2009). Identification and characterization of adult stem cells from human orbital adipose tissue. *Ophthalmic Plastic and Reconstructive Surgery*.

[42] Lubin JR, Albert DM, Weinstein M (1980). Sixty-five years of sympathetic ophthalmia. A clinicopathological review of 105 cases (1913–1978). *Ophthalmology*.

[43] Kikkawa DO, Pornpanich K, Cruz RC, Levi L, Granet DB (2002). Graded orbital decompression based on severity of proptosis. *Ophthalmology*.

[44] Chang EL, Bernardino CR, Rubin PAD (2003). Transcaruncular orbital decompression for management of compressive optic neuropathy in thyroid-related orbitopathy. *Plastic and Reconstructive Surgery*.

[45] Graham SM, Brown CL, Carter KD, Song A, Nerad JA (2003). Medial and lateral orbital wall surgery for balanced decompression in thyroid eye disease. *Laryngoscope*.

[46] Rundle FF, Wilson CS (1945). Development and course of exophthalmos and ophthalmoplegia in Graves' disease with special reference to the effect of thyroidectomy. *Clinical Science*.

[47] Baldeschi L, Wakelkamp IMMJ, Lindeboom R, Prummel MF, Wiersinga WM (2006). Early versus late orbital decompression in Graves' orbitopathy: a retrospective study in 125 patients. *Ophthalmology*.

